# Non-invasive detection of somatic mutations using next-generation sequencing in primary central nervous system lymphoma

**DOI:** 10.18632/oncotarget.18325

**Published:** 2017-06-01

**Authors:** Maxime Fontanilles, Florent Marguet, Élodie Bohers, Pierre-Julien Viailly, Sydney Dubois, Philippe Bertrand, Vincent Camus, Sylvain Mareschal, Philippe Ruminy, Catherine Maingonnat, Stéphane Lepretre, Elena-Liana Veresezan, Stéphane Derrey, Hervé Tilly, Jean-Michel Picquenot, Annie Laquerrière, Fabrice Jardin

**Affiliations:** ^1^ Department of Hematology, Cancer Center Henri Becquerel, 76000 Rouen, France; ^2^ INSERM U1245, Cancer Center Henri Becquerel, Institute of Research and Innovation in Biomedicine (IRIB), University of Normandy, UNIVROUEN, 76000 Rouen, France; ^3^ INSERM U1245 and Hôpital Charles Nicolle, NeoVasc Team, University of Normandy, UNIVROUEN, CHU-Hôpitaux de Rouen, 76031 Rouen, France; ^4^ Department of Neuropathology, Hôpital Charles Nicolle, Normandy Center for Genomic and Personalized Medicine, CHU-Hôpitaux de Rouen, 76031 Rouen, France; ^5^ Department of Pathology, Cancer Center Henri Becquerel, 76000 Rouen, France; ^6^ Department of Neurosurgery, Hôpital Charles Nicolle, CHU-Hôpitaux de Rouen, 76031 Rouen, France

**Keywords:** primary central nervous system lymphoma, circulating cell-free tumor DNA, somatic mutation, liquid biopsy, next-generation sequencing

## Abstract

**Purpose:**

Primary central nervous system lymphomas (PCNSL) have recurrent genomic alterations. The main objective of our study was to demonstrate that targeted sequencing of circulating cell-free DNA (cfDNA) released by PCNSL at the time of diagnosis could identify somatic mutations by next-generation sequencing (NGS).

**Patients and Methods:**

PlasmacfDNA and matched tumor DNA (tDNA) from 25 PCNSL patients were sequenced using an Ion Torrent Personal Genome Machine (Life Technologies^®^). First, patient-specific targeted sequencing of identified somatic mutations in tDNA was performed. Then, a second sequencing targeting *MYD88* c.T778C was performed and compared to plasma samples from 25 age-matched control patients suffering from other types of cancer.

**Results:**

According to the patient-specific targeted sequencing, eight patients (32% [95% CI 15-54%]) had detectable somatic mutations in cfDNA. Considering *MYD88* sequencing, six patients had the specific c.T778C alteration detected in plasma. Using a control group, the sensitivity was 24% [9-45%] and the specificity was 100%. Tumor volume or deep brain structure involvement did not influence the detection of somatic mutations in plasma.

**Conclusion:**

This pilot study provided evidence that somatic mutations can be detected by NGS in the cfDNA of a subset of patients suffering from PCNSL.

## INTRODUCTION

Primary central nervous system lymphoma (PCNSL) accounts for 1% of adult lymphoma and 5% of primary central nervous system (CNS) tumors [1, 2]. PCNSL differs from nodal diffuse large B cell lymphoma (DLBCL) by displaying a more aggressive behavior and a worse prognosis [3]. In the past decade, nucleic acid sequencing through next-generation sequencing (NGS) has revealed genomic alterations in PCNSL [4–9]. Among the nuclear factor-kappa B (NF-κB) pathway genes, *MYD88* and *CD79B* alterations have been identified in 40% to 80% of all cases [7, 10–12], and they may confer sensitivity to the B cell receptor (BCR) signaling pathway inhibitor [13, 14]. Thus, molecular analysis is fast becoming a topic of major interest for patient care. In this context, deep-sequencing of tumor DNA could constitute a routine test at the time of diagnosis in patients suffering from lymphomas [15], particularly in PCNSL cases. Indeed, PCNSL samples are obtained from surgical or stereotactic biopsies at the time of diagnosis. A minimally invasive method that helps with the molecular diagnosis of targetable alterations could represent a valuable advance in PCNSL management. Tumor genomic DNA may be obtained in the blood of patients suffering from cancer. Circulating cell-free DNA (cfDNA) composed of nucleic acid fragments circulating in human fluids, such as plasma, could be derived from tumor cells [16–18]. Plasma circulating cell-free tumor DNA (ctDNA) contains fragments of the tumor genome with somatic alterations [19] and has already been validated in patients affected by nodal DLBCL [20].

The aim of the study was to assess NGS as a minimally invasive approach to detect PCNSL somatic mutations in ctDNA. For this purpose, we performed NGS to compare the pattern of somatic mutations in plasma ctDNA and in tumors and its diagnostic performance at the time of the initial diagnosis in patients suffering from PCNSL. Secondary objectives were to identify parameters that could influence ctDNA release in plasma and to evaluate high initial cfDNA concentration as a risk factor for survival.

## RESULTS

### Clinical and histological features

NGS was performed for 25 patients. The mean patient age was 67 (range, 49 to 87). Twenty patients (80%) had stereotactic biopsies alone and 5 patients (20%) underwent partial tumor resection. Sixteen patients received an initial body PET/CT and the remaining nine patients had chest/abdomen/pelvis CT scans. No extra-axial malignant lesion was found. Bone marrow biopsies (n=20) or aspirates (n=5) did not identify any pathological bone marrow involvement. All PCNSL were EBV negative. All patients were given corticosteroids at a dose between 1 and 1.5mg/kg, right after neurosurgery and before blood collection. Details of the PCNSL cohort are provided in Table 1.

**Table 1:** The clinical, tumor and biological characteristics of the PCNSL cohort at the time of blood collection, (n=25)

	All PCNSL	Mutation(s) detected in cfDNA		
	n=25	Yes, n=8	No, n=17	p Value^a^
Gender				
Female	10 (40%)	1 (13%)	9 (53%)	0.09^b^
Male	15 (60%)	7 (87%)	8 (47%)	
Age (years), mean±standard deviation	67 ±9.1	65 ±9.3	68 ±9.1	0.4^c^
MSKCC score				
1	1 (4%)	1 (13%)	0	0.1^d^
2	15 (60%)	6 (74%)	9 (53%)	
3	9 (36%)	1 (13%)	8 (47%)	
Neurosurgical intervention				
Stereotactic biopsy	20 (80%)	7 (88%)	13 (76%)	0.99^b^
Tumor resection	5 (20%)	1 (12%)	4 (24%)	
Tumor				
Percentage of tumor cells				
25-50%	7 (28%)	8 (100%)	4 (24%)	0.5^d^
>50%	18 (72%)	-	13 (76%)	
Deep brain structure involvement^e^	10 (40%)	2 (25%)	8 (47%)	0.4^b^
Volume (cm3), mean ±sd	15.8 ±15	16 ±7	15.8 ±17	0.9^c^
Plasmatic [cfDNA] (ng/ml), mean ±sd	64 ±40	64 ±40	64 ±4.4	0.9^c^
Time between biopsy and blood collection (day)	23 ±11	22 ±8	24 ±12	0.7^c^

*^a^*Comparisons between patients with and without detected mutations in cfDNA.

*^b^*The Fisher exact test.

*^c^*Student's unpaired t-test.

*^d^*Pearson's chi-squared test.

*^e^*Defined as a tumor located in the periventricular territory.

cfDNA: circulating cell free DNA; MSKCC: Memorial Sloan Kettering Cancer Centre.

### PCNSL tDNA sequencing

**Figure 1:**
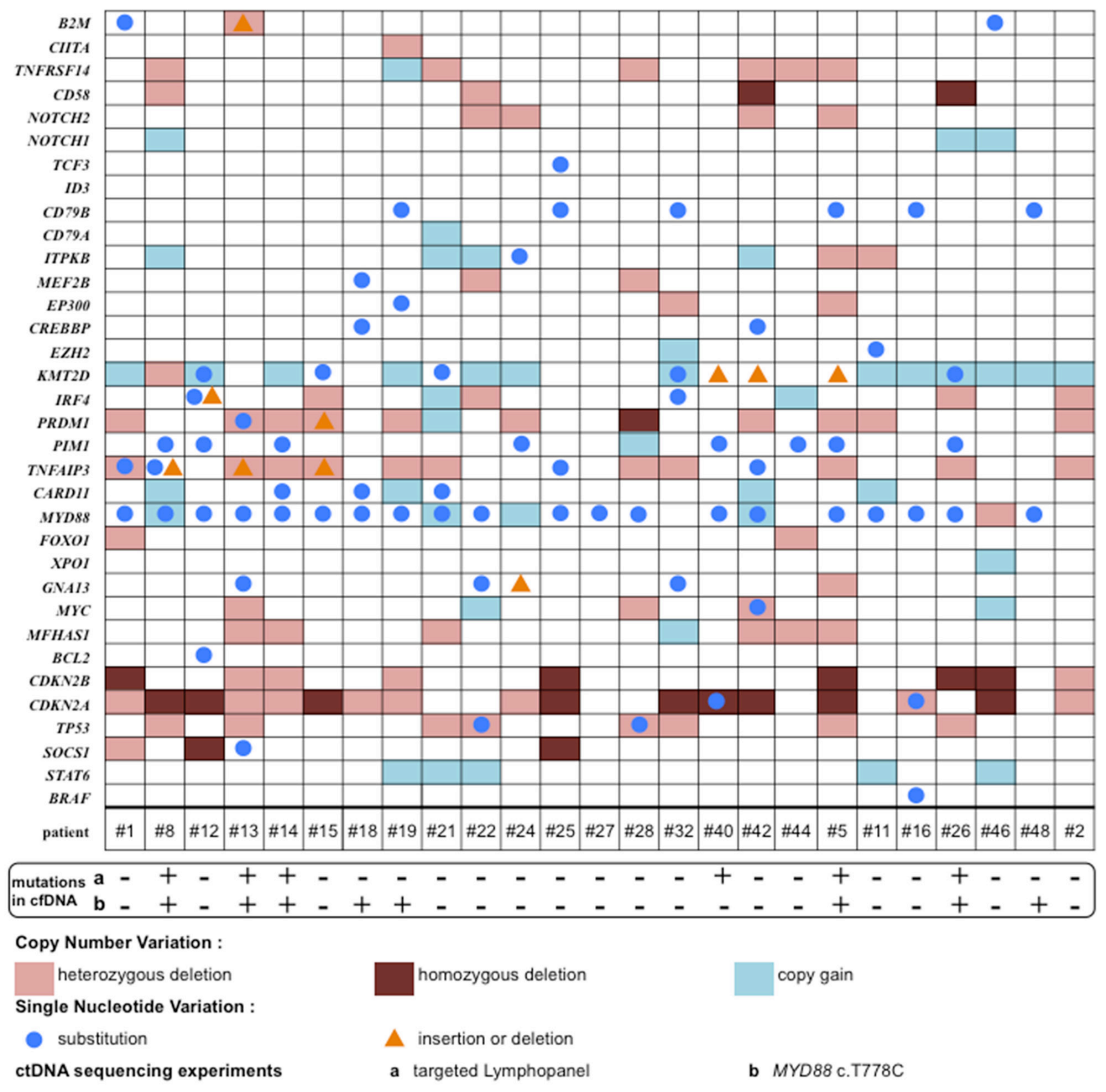
Mutational profile of tumor genomic alterations per patient (n=25) The alterations are function-altering variants (SNV, insertion or deletion) and copy number variants (copy gain, heterozygous or homozygous deletion) detected per patient on the horizontal axis and per gene on the vertical axis for the entire PCNSL cohort. *MYD88* and *CDKN2A* were the most commonly affected genes with regard to SNV and heterozygous or homozygous deletions, respectively.

**Figure 2:**
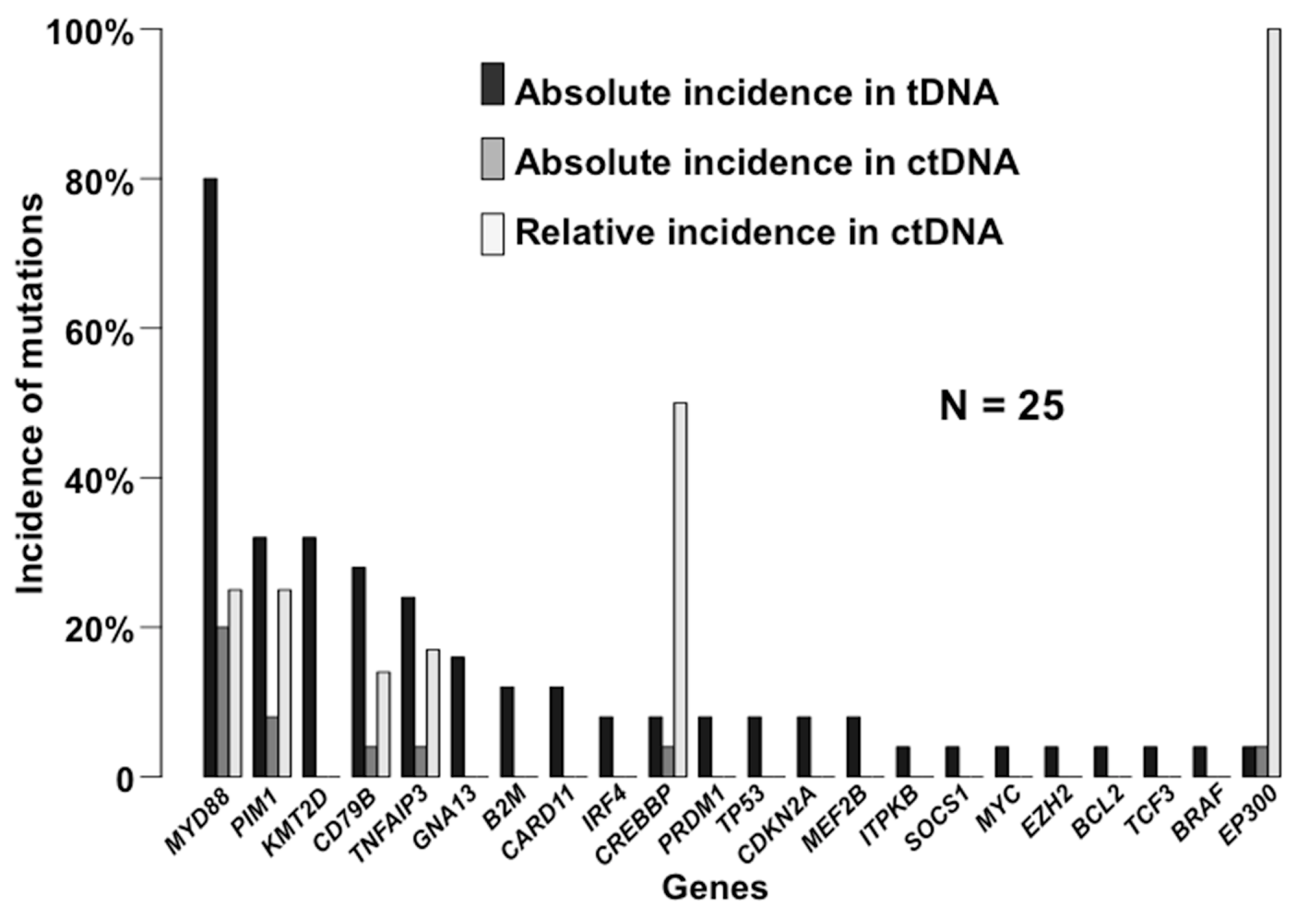
The incidence of somatic mutations in PCNSL identified by Lymphopanel sequencing, and in the matched ctDNA according to the targeted panel sequencing Mutation frequencies are expressed per gene in this stacked histogram. Black bars represent altered genes for PCNSL, dark gray bars represent the absolute proportion in the ctDNA, and light gray bars represent the relative proportion in the ctDNA.

The mean tumor DNA (tDNA) concentration was 57 μg/mL [95% CI 57.5 – 157 μg/mL]. All samples harbored an alteration that was identified using the Lymphopanel: either function-altering variants or copy number variation (CNV) (Figure 1). The mutational profile was consistent with previously published data [4, 5, 7, 10], and, as expected, *MYD88* (80%)*, PIM1* (32%) and *CD79B* (28%) were the most commonly mutated genes (Figure 2).

### cfDNA sequencing: targeted panel results

**Figure 3:**
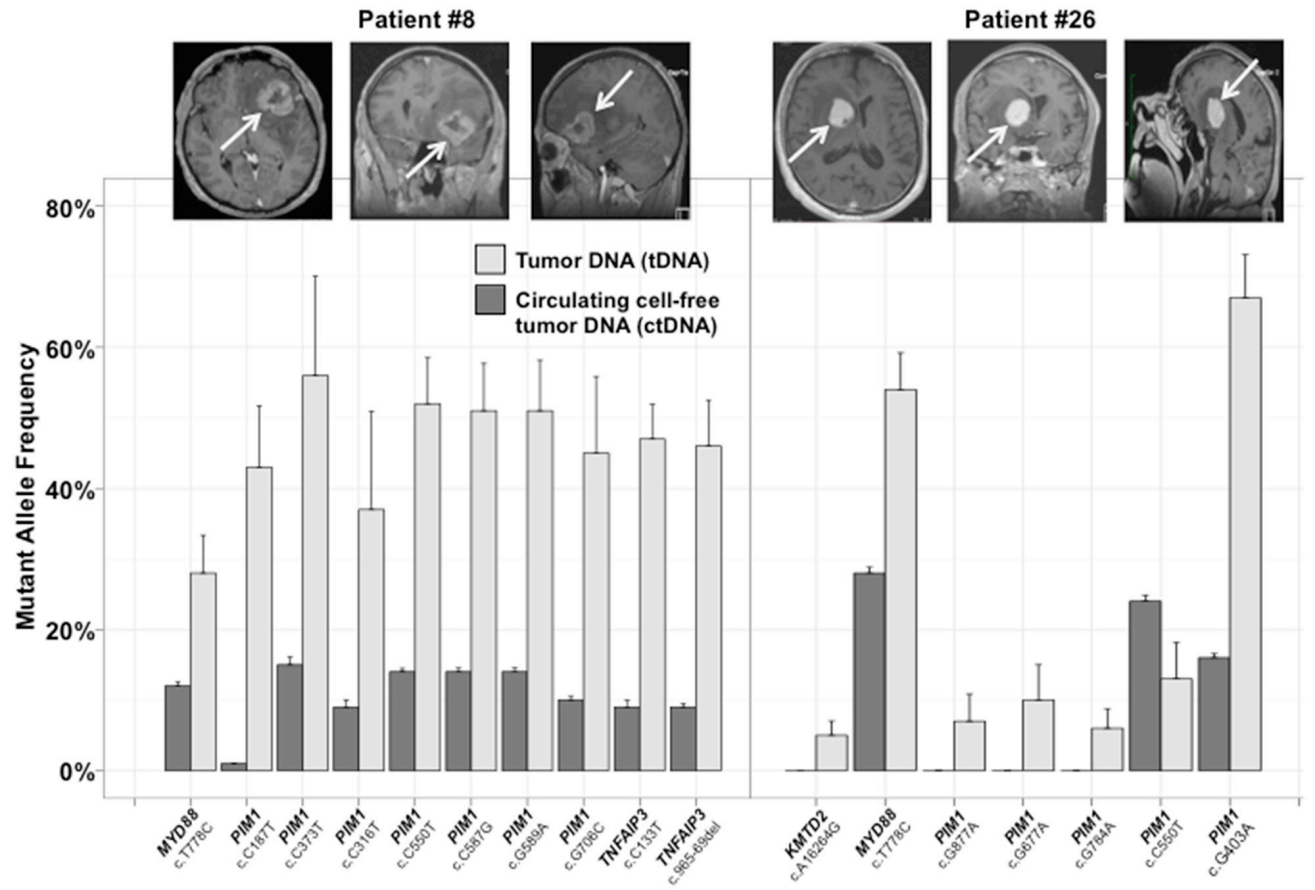
Two representative examples of the mutant allele frequencies (MAF) identified in tumor and matched plasma ctDNA The MAF corresponds to the proportion of mutated reads compared to all reads for one specific genomic location. The absence of a histogram bar indicates that no mutation was identified.

In eight of twenty-five patients (32% [95% CI 15% - 54%]), somatic mutations in ctDNA were detected by sequencing with the targeted panel. The mutational pattern and altered genes frequencies found in the plasma differed from the matched PCNSL (Figure 2). Two patients (8%) harbored the same mutational profile in their tumors and cfDNA, with *MYD88*, and *TNFAIP3* mutations and somatic hyper mutation affecting *PIM1* in one case (Figure 3). Performing the targeted cfDNA panel increased the coverage depth compared to tDNA sequencing (Supplementary Table 1): the mean number of reads for the altered genes in ctDNA was 12550x [95% CI 9270x – 15837x] versus 185x [160x – 210x] in the tumors (p<0.001).

### Diagnostic performance of circulating *MYD88* c.T778C

*MYD88* c.T778C was detected in blood samples from six different patients (35% [95% CI 12.5% – 58.0%], Table 2). The mutant allele frequency (MAF) of the *MYD88* c.T778C was significantly lower in the ctDNA compared to the tumors: mean MAF 4.7% [0.5% – 9%] versus 47% [35.5% – 57.6%] respectively, p<0.001.

**Table 2:** Case – control study of *MYD88* c.T778C detection by NGS

	PCNSL								Control					
Patient no		Tumor DNA				Circulating tumor DNA					Circulating tumor DNA			
	Age (y)	*MYD88 c.T778C*	Percentage of tumor cell	MAF (%)	Reads number	MAF (%)	Reads number	*MYD88 c.T778C*	Age (y)	Histology	MAF (%)	Reads number	*MYD88 c.T778C*	Concentration (ng/ml)
#13	49	**Pos**	>50%	60.09	134/223	0.6	143/23708	**Pos**	31	GBM	<0.5	74/48876	Neg	41.2
#28	53	**Pos**	>50%	25.56	23/90	<0.5	18/8958	Neg	52	GBM	<0.5	48/42009	Neg	65.2
#22	53	**Pos**	>50%	43.92	159/362	<0.5	24/23898	Neg	55	GBM	<0.5	61/40962	Neg	54.4
#16	57	**Pos**	>50%	43.48	70/161	<0.5	49/42713	Neg	58	GBM	<0.5	56/47997	Neg	43.2
#44	60	Neg	>50%	-	-	<0.5	33/22877	Neg	59	CRC	<0.5	70/42143	Neg	128
#12	61	**Pos**	>50%	47.5	76/160	<0.5	62/20731	Neg	61	CRC	<0.5	94/53803	Neg	53.6
#19	61	**Pos**	25%	26.58	21/79	<0.5	16/17867	Neg	61	GBM	<0.5	91/57498	Neg	20.1
#48	62	**Pos**	>50%	7.58	21/277	<0.5	6/5848	Neg	62	CRC	<0.5	56/50644	Neg	69.2
#8	63	**Pos**	>50%	28.48	94/330	11.2	5146/46074	**Pos**	62	GBM	<0.5	45/38929	Neg	54.8
#1	63	Neg	>50%	-	-	<0.5	36/26209	Neg	63	CRC	<0.5	78/49144	Neg	53.2
#27	63	**Pos**	25%	15.6	17/109	<0.5	59/31514	Neg	63	CRC	<0.5	54/50353	Neg	52.4
#18	64	**Pos**	>50%	44.03	70/159	<0.5	23/25640	Neg	65	GBM	<0.5	78/59889	Neg	25.6
#26	65	**Pos**	>50%	54	189/350	28.7	14356/49954	**Pos**	65	GBM	<0.5	67/59691	Neg	40.8
#2	67	Neg	>50%	-	-	<0.5	9/14377	Neg	67	CRC	<0.5	63/29102	Neg	120
#25	68	**Pos**	25-50%	67.74	147/217	<0.5	39/32563	Neg	68	CRC	<0.5	65/56826	Neg	44.8
#42	68	**Pos**	>50%	24.1	60/249	<0.5	64/60828	Neg	69	CRC	<0.5	84/56066	Neg	55.6
#15	69	Neg	>50%	-	-	<0.5	74/42979	Neg	71	GBM	<0.5	57/28504	Neg	54
#5	71	**Pos**	25-50%	91.12	359/394	7.7	3401/44285	**Pos**	72	CRC	<0.5	48/34430	Neg	72
#46	71	Neg	25-50%	-	-	<0.5	42/38638	Neg	72	CRC	<0.5	53/57963	Neg	57.6
#24	74	Neg	>50%	-	-	<0.5	49/27303	Neg	75	GBM	<0.5	70/44478	Neg	35.52
#21	77	**Pos**	>50%	67.31	313/465	<0.5	48/27004	Neg	75	GBM	<0.5	81/50177	Neg	43.6
#11	78	Neg		-	-	<0.5	82/35998	Neg	79	CRC	<0.5	53/32229	Neg	117.6
#14	82	**Pos**	25-50%	67.27	37/55	0.5	91/18056	**Pos**	81	GBM	<0.5	46/52808	Neg	42.8
#40	82	**Pos**	25%	97.43	341/350	0.5	131/25528	**Pos**	82	CRC	<0.5	59/47827	Neg	39.2
#32	87	Neg	>50%	-	-	<0.5	26/37256	Neg	85	CRC	<0.5	60/49963	Neg	55.2

CRC: colorectal cancer; GBM: glioblastoma multiforme; MAF: mutant allele frequency; neg: negative; pos: positive; y: years.

**Figure 4:**
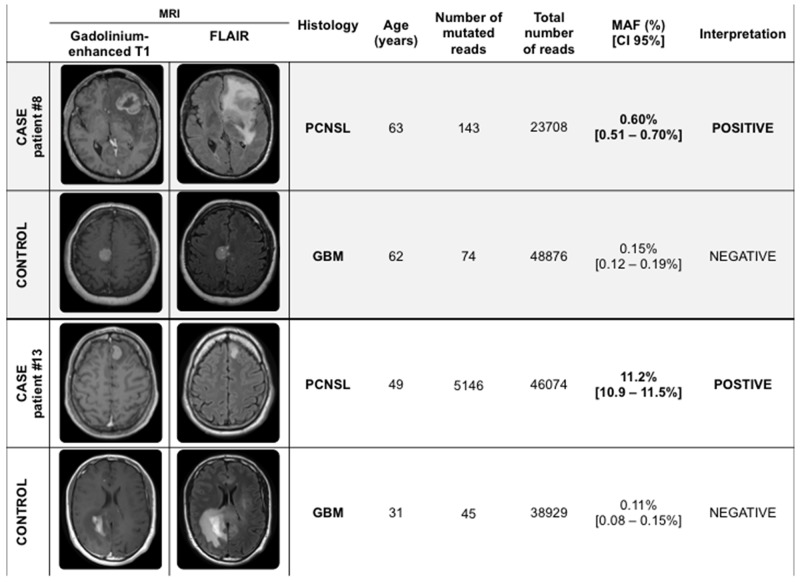
Two representative case-control examples of the detection of *MYD88* c.T778C in plasma samples by NGS

All plasmas from the control group contained cfDNA. No mutation was detected in the cfDNA from the control group (0/25), according to our threshold levels (Table 2). Among the six patients with *MYD88* c.T778C in plasma, NGS made it possible to distinguish PCNSL from glioblastoma (GBM) in 4 cases (Figure 4, Table 2). Overall, NGS performed on cfDNA had a high positive predictive value (100%) and a high specificity (100%). The negative predictive value was 56.8% [95% CI 42.2% – 71.5%] and the sensibility was at 24% [95% CI 9% – 45%].

### Secondary objectives

The detection of mutations in ctDNA was independent from the clinical or tumor characteristics (Table 1). Importantly, the surgical procedure (biopsy versus resection) did not influence the detection of mutations in ctDNA or the concentration of cfDNA (data not shown).

All PCNSL plasmas contained cfDNA with a mean concentration of 64 ng/mL [95% CI 48 – 80 ng/mL]. No association was found between the tumor volume and plasma cfDNA concentration: R-squared was 0.01, p=0.68. Deep brain structures involvement (n=10) did not seem to influence the plasma cfDNA concentration: mean concentration of 72 ng/mL versus 60 ng/mL for the remaining 15 tumors, p=0.51.

For the cohort of PCNSL patients, the median follow-up was 21.5 months (range, 2-60 months). The median overall survival was 20.5 months. Univariate analyses showed that the Memorial Sloan Kettering Cancer Center (MSKCC) score >2 correlated negatively with OS, HR 2.6 [1.04-6.56], p=0.0378. The presence of the *MYD88* c.778T>C mutation tend to be correlated with a shorter OS, as previously reported [21], HR 2.55 [0.78-8.36], p=0.1. Multivariate analysis showed that only the MSKCC score >2 remained statistically significant for OS, HR 2.9 [1.11-7.63], p=0.028. In addition, ctDNA concentration ≥64 ng/ml showed a non-significant trend to confer a survival disadvantage: HR 1.9 [95% CI 0.68-5.23], p=0.21 (Table 3).

**Table 3:** Univariate and multivariate analyses of risk factors for overall survival

	Univariate analysis		Multivariate analysis	
	HR [95% CI]	p Value^a^	HR [95% CI]	p Value
MSKCC score >2	2.6 [1.04-6.56]	0.0378 *	2.9 [1.11-7.63]	0.028 *
[ctDNA] ≥64 ng/ml	1.9 [0.68-5.23]	0.21	-	-
*MYD88* c.778T>C	2.55 [0.78-8.36]	0.1 ^†^	2.99 [0.88-10.27]	0.08

ctDNA: circulating cell-free tumor DNA; CI: confidence interval; HR: hazard ratio; MSKCC: Memorial Sloan Kettering Cancer Centre; OS: overall survival.

^a^ The log rank test.

*, 0.05; †, 0.1

## DISCUSSION

In this study, we demonstrated that tumor-derived somatic mutations could be identified in plasma of one third of PCNSL patients, thereby proving that, similar to other localizations, the detection of ctDNA in PCNSL is possible. The detection rate (8/25, 32%) was similar to those of other studies that included patients suffering from primary brain tumors: 9/33 (27%) in a cohort of GBM patients [22], and 8/41 (19.5%) in a cohort of medulloblastoma and glioma patients [23]. A key point for discussion of this diagnostic test is its low sensitivity (24%). The true positive rate is dependent on two major parameters: the concentration of cfDNA and the method used for detecting mutations. Regarding the first point, cfDNA concentrations were low in the plasma from both PCNSL patients (mean 64 ng/ml) and GBM patients (mean 44 ng/ml). Nevertheless, those levels are higher than previously published, with a mean of 14 ng/ml in the plasma from 14 patients suffering from high grade gliomas [24]. In this study, Boisselier et al. found that tumor with contrast enhancement on MRI had a higher plasma cfDNA concentration. As all of PCNSL and GBM in the cohort experienced contrast enhancement, this parameter could account for our higher cfDNA concentration. Moreover, clinical or histological parameters that could influence cfDNA release in plasma remain unclear in the specific population of PCNSL patients. Based upon murine models it has been emphasized that the presence of nucleic acid in the blood could depend on macrophage activity in the tumors [25, 26]. Hohaus et al. have shown that histological signs of necrosis in Hodgkin's lymphoma are associated with increased plasma cfDNA: median 42.1 ng/ml versus 21.5 ng/ml, p=0.03 [27]. In our cohort, these parameters could not be studied due to the small tumor size sampling with subsequent underestimation of tumor necrosis. Tumor burden (e.g., the overall tumor volume or advanced stage of disease) has been shown to influence the detection of tumor-derived alterations in blood cfDNA for nodal DLBCL [27]. Here, we demonstrated that, in contrast to nodal lymphoma, the release of cfDNA in plasma seems to be independent of tumor volume (R-squared = 0.01) or cerebral location, as it has been already reported in low-grade glioma [24]. Regarding the high false negative rate, it could be explained by the administration of corticosteroids right after the surgical procedure and before blood collection. Parameters that could influence the plasma release and detection of cfDNA remain unclear in this setting and should be investigated in further studies.

Baraniskin et al. have already shown the potential value of microRNAs in cerebrospinal fluid (CSF) concerning the diagnosis, treatment monitoring and follow-up of PCNSL patients [28, 29]. De Mattos-Arruda et al. have also shown that the sensitivity for detecting ctDNA was higher in CSF than in plasma (58% versus 0%, p=0.0006) in a cohort of patients suffering from malignant tumor restricted to the CNS. However, it appeared that the required CSF volume was at least 1-2 ml, which could be difficult to collect by lumbar puncture. In the study of De Mattos-Arruda et al., CSF was obtained using cerebral shunts or at the time of the patient death as the first step of autopsy. Because our study aimed at finding a minimally invasive biomarker that could be collected at the time of diagnosis and several times at follow-up, we did not explore tumor-derived genomic alterations in CSF. In future studies, it would be of interest to compare the mutational pattern of ctDNA in plasma and in CSF in a subgroup of patients who may have a lumbar puncture with adequate CSF samples.

Given the method to detect somatic mutations in plasma ctDNA, targeted sequencing by NGS has already shown its utility in clinical practice and translational research for several solid tumors or lymphoma [22, 30–32]. In this pilot study, our method was capable of detecting *MYD88* c.T778C in tDNA and matched ctDNA with good concordance in a subset of patients. The Lymphopanel developed by our laboratory for nodal DLBCL remains informative in PCNSL cases. It could also be improved by the addition of recurrent mutations found in PCNSL, such as *TBLXR1, ETV6, EBF1* or *IRF2BP2* [7] or by the addition of the VDJ rearrangement [20]. A reduced PCNSL-dedicated panel that would include *MYD88*, *CD79B*, *PIM1*, *BTG2* and *TBLXR1* could be of major interest to improve the sensitivity of detection of ctDNA in plasma.

Our results also underscore that mutational profiles mostly differ between tDNA and ctDNA. This discordance has already been emphasized in early-stage lung cancer patients. Jamal-Hanjani et al. have shown that the low level of MAF in tDNA could account for the mutational heterogeneity between tumor and blood samples. In our cohort, mutations in tDNA with MAF <10% were never detect in cfDNA. Conversely, we found no association between detection in ctDNA and tDNA for MAFs over 10%. In this setting, tumor sampling by biopsy may contain an overestimated proportion of mutations, which are heterogeneous in the whole tumor. In addition to tumor heterogeneity, this result may reflect cfDNA alterations and subsequent distinct PCR efficacy between tumor and cfDNA. Furthermore, the use of a selected set of primers to analyze cfDNA, compared to the full set of primers used to analyze tDNA may change the PCR conditions and account for these results.

We wished to evaluate the diagnostic performance of detecting the circulating *MYD88* c.T778C in plasma. The circulating *MYD88* c.T778C mutation has been described in patients suffering from Waldenström macroglobulinemia (WM) or IgM monoclonal gammopathy of undetermined significance (MGUS) [33, 34]. Xu et al. have already shown that the circulating *MYD88* c.T778C was not identified in the peripheral blood by allele-specific PCR in 40 healthy subjects. For this reason and due to ethical considerations, sequencing of cfDNA from healthy subjects was not performed in this study. Nevertheless, *MYD88* c.T778C was found neither in the plasma of GBM patients nor in colorectal adenocarcinoma (CRC) patients. These results are in agreement with those of Fukumura et al. They have suggested that Myd88 p.L265P could be present in peripheral blood mononuclear cells as a preliminary event in mutated *MYD88* PCNSL lymphoma genesis [4]. The extracerebral, especially medullary, origin of pathological clones of PCNSL remains an unsolved problem. In this study, the bone marrow was only examined at the microscopic level. No patients displayed a pathological involvement. It would be of interest to compare the profiles of lymphocyte phenotypes, clonality and determine the presence of recurrent mutations (involving *MYD88*, *CD79B* and *PIM1* genes) in the tumor, plasma and bone marrow.

Finally, the concentration of ctDNA at the time of the diagnosis appeared to correlate to clinical outcomes, as previously reported in nodal DLBCL [35]. Our results are exploratory and should be interpreted with caution because of the small size of the study population. In addition, although all patients received high doses of methotrexate, the treatment regimens mostly differed in the PCNSL cohort. Despite these limiting points, a high concentration of ctDNA could suggest a poor prognosis. One explanation would be its correlation to tumor aggressiveness such as total volume, necrosis volume and tumoral perfusion. The prognostic impact of ctDNA concentration in PCNSL should be explored in a larger cohort and should be correlated to MRI and histological parameters of necrosis.

The plasma of patients suffering from PCNSL contains detectable somatic genomic alterations released from tumor cells. This study provides evidence that the liquid biopsy concept, as a minimally invasive diagnosis test, is achievable for a subset of these patients. Furthermore, NGS may be used as a molecular diagnostic method prior to delivering targeted therapies, particularly BCR inhibitors in the case of *MYD88* mutated tumors [13, 14]. The MAF of detected mutations is also a promising quantitative biomarker for the response to treatment, patient follow-up and early detection of relapse [36].

## MATERIALS AND METHODS

### Patients, and tumor and plasma samples

HIV negative patients suffering from newly diagnosed DLBCL PCNSL [37] between 2008 and 2014 were selected for this study. Shortly after surgery, tumor samples were processed for histological diagnosis, and a fragment was stored at -80°C until use. Cases with less than 25% tumor content were excluded from the analysis. Blood samples were obtained postoperatively and before the administration of first-line treatment with methotrexate. Patients with extra-axial lesions defined by histologically proven cancer within two years of their study inclusion were excluded. Patients with a previous history of lymphoma and/or Waldenström macroglobulinemia (WM) and/or IgM monoclonal gammopathy of undetermined significance (MGUS), at any time, were also excluded. According to local guidelines, the staging procedure was performed for all patients between the histological diagnosis and the first line treatment, within the month of blood collection. The staging procedure included a body F18-fluorodeoxyglucose positron emission tomography/computed tomography (body PET/CT) and a bone marrow biopsy, when feasible. An internal review board approved the study (reference No. 1504B).

### DNA extraction and sequencing

Tumor PCNSL DNA was extracted and sequenced using a dedicated Lymphopanel (Supplementary Table 2) as previously reported in a cohort of nodal DLBCL [38]. An Ion Torrent Personal Genome Machine (PGM, Life Technologies^®^, Carlsbad, California, United States of America) was used. Amplified libraries (Ion AmpliSeq^®^ Library Kit 2.0) were submitted to emulsion PCR using the Ion OneTouch^®^ 200 Template Kit (Life Technologies^®^) with the Ion OneTouch^®^ System (Life Technologies^®^) according to the manufacturer's instructions. The templated Ion Sphere^®^ particles were enriched with the Ion OneTouch^®^ Enrichment System and then loaded and sequenced on an Ion 316™ v2 Chip (Life Technologies^®^). A pool of oligonucleotide primers was selected among the 872 pairs provided by Life Technologies^®^. Reads were aligned to hg19 genome using the Torrent Mapping Alignment Program (TMAP). TMAP is a tool combining multiple mapping algorithms for Ion Torrent^®^ data. It uses three core algorithms: Burrows-Wheeler Alignment (BWA) tool for short read [39], BWA for long read [40] and a modified Sequence Search and Alignment by Hashing Algorithm (SSAHA) version [41]. TMAP follows a two-stage approach, with a set of algorithms and associated settings for each stage. If there is no mapping for a read by applying the algorithms in the first stage, then the algorithms in the second stage are applied. Furthermore, TMAP allows applying penalties using flowgram information, making it particularly efficient in regions enriched with homopolymers. Only non-synonymous single nucleotide variations (SNV) with a quality score >22 were retained as acquired somatic mutations. The limit of detection (LOD) and the limit of quantification (LOQ) were at 1% of MAF, as set by the manufacturer. Copy number variation (CNV) analysis was performed using the ONCOCNV package [42].

Blood samples were obtained postoperatively and before the first-line treatment. Plasma was extracted from EDTA-anticoagulated blood samples by centrifuging once at 2000 g (10 min) at 4°C and stored at -80°C until use. cfDNA was extracted from 1 ml of EDTA plasma using the QIAamp® Circulating Nucleic Acid Kit (Qiagen®, Hilden, Germany) with the QIAvac 24 Plus vacuum manifold, following the manufacturer's instructions. Extracted cfDNA from each plasma sample was eluted in a volume of 40 μl. cfDNA concentrations were measured using a fluorometric assay (Qubit® dsDNA HS Assay Kit, Life Technologies®, Carlsbad, California, United States of America). According to the tDNA mutation pattern and to increase the detection rate, matched cfDNA was subsequently sequenced using selected primers (called the ‘targeted panel’) as previously described [43]. The procedure to create libraries and to sequence amplicons was the same as that used for tDNA, except for a 314™ v2 chip.

### Case-control study

To assess the diagnostic performance of NGS with cfDNA, an age-matched case-control study was performed. *MYD88*, which is known to be the most recurrent gene in PCNSL [4, 21, 44] was selected for this purpose. We surmised that the most common mutation would be *MYD88* c.778T>C. Targeted sequencing was performed on serial dilutions of known mutant DNA (from the OCI-Ly3 cell line, which is homozygous for c.778T>C) in a wild-type sample. We tested 0%, 0.05%, 0.1%, 0.5% and 1% dilutions of the mutant allele, and sequencing was performed as it was for plasma cfDNA (twice in triplicate). For this specific alteration we were able to determine the detection and quantification limits in plasma down to 0.5% (Supplementary Table 3), as calculated by Armbruster et al. [45]. Age-matched control plasmas were selected among patients suffering from histologically proven GBM or CRC. GBM was chosen because of its well described mutational profile that does not include mutations of *MYD88* [46] and because of its high frequency among primary brain tumors, leading to a recurrent differential diagnosis of PCNSL. The second group was composed of patients suffering from metastatic CRC, which are known to have a high cfDNA concentration. All patients were free of hematological disorder after an extensive evaluation that comprised a body scan, blood count and no previous history of hematological malignancy. Blood collection, cfDNA extraction and sequencing experiment were the same as for PCNSL cases. Written informed consent was obtained. Thus, the sensitivity, specificity, and negative and positive predictive value of detecting *MYD88* c.T778C in ctDNA at the time of diagnosis were estimated.

### Secondary objectives

MSKCC score, tumor volume, type of neurosurgery, percentage of tumor cells, deep brain involvement and cfDNA concentration in plasma were compared between patients with ctDNA detected in plasma and patients without detected ctDNA. Tumor volume was estimated using postcontrast T1-weighted MRI. Section thickness was between 3 and 5 mm. The tumor area, defined as contrast enhancement, was delineated on each slide in the axial section. The volume was estimated using image-processing software (OsiriX^®^, Pixmeo, Geneva, Switzerland). Deep brain involvement was defined by tumor involvement of subcortical structures on T1-weighted MRI.

Finally, an exploratory survival analysis was performed. We investigated three risk factors for overall survival (OS): the MSKCC score, *MYD88* c.778T>C mutation in tDNA and a high concentration of ctDNA in plasma at time of the diagnosis, defined by a concentration equal or higher than the mean in the PCNSL cohort. Survival was calculated in months from the date of biopsy to the date of death for any reason.

### Statistical methods

Statistical analyses were performed using R software (R Foundation for Statistical Computing, Vienna, Austria). Clinical and biological data were compared using Student's unpaired t-test for continuous variables, and the Fisher exact test or Pearson's chi-squared test was used for categorical variables, as appropriate. Associations between continuous variables were estimated with R-squared using the linear regression model. The log-rank test investigated OS differences among groups. Univariate analyses were first performed for each variable. Then, variables that were significantly correlated to OS (p-value equal or lower than 0,10) were selected for the multivariate analysis, performed with the Cox regression model. In the multivariate analysis the alpha risk threshold was set at 5%.

## SUPPLEMENTARY MATERIALS AND TABLES





## Abbreviations

AS-PCR: allele specific polymerase chain reaction
BCR: B cell receptor
BWA: Burrows-Wheeler alignment
CNS: central nervous system lymphoma
CNV: copy number variation
CRC: colorectal carcinoma
DLBCL: diffuse large B cell lymphoma
cfDNA: circulating cell-free DNA
ctDNA: circulating tumor DNA
tDNA: tumor DNA
EBV: Epstein Bar virus
GBM: glioblastoma multiforme
LOD: limit of detection
LOQ: limit of quantification
MAF: mutant allele frequency
MGUS: monoclonal gammopathy of undetermined significance
NGS: next-generation sequencing
PCNSL: primary central nervous system lymphoma
PET/CT: positron emission tomography/computed tomography
SNV: single nucleotide variation
SSAHA: Sequence Search and Alignment by Hashing Algorithm
TMAP: Torrent Mapping Alignment Program
WM: Waldenström macroglobulinemia.

## References

[1] Miller DC, Hochberg FH, Harris NL, Gruber ML, Louis DN, Cohen H (1994). Pathology with clinical correlations of primary central nervous system non-Hodgkin's lymphoma. The Massachusetts General Hospital experience 1958-1989. Cancer.

[2] Behin A, Hoang-Xuan K, Carpentier AF, Delattre JY (2003). Primary brain tumours in adults. Lancet.

[3] Ferreri AJ, Blay JY, Reni M, Pasini F, Spina M, Ambrosetti A, Calderoni A, Rossi A, Vavassori V, Conconi A, Devizzi L, Berger F, Ponzoni M (2003). Prognostic scoring system for primary CNS lymphomas: the International Extranodal Lymphoma Study Group experience. J Clin Oncol.

[4] Fukumura K, Kawazu M, Kojima S, Ueno T, Sai E, Soda M, Ueda H, Yasuda T, Yamaguchi H, Lee J, Shishido-Hara Y, Sasaki A, Shirahata M (2016). Genomic characterization of primary central nervous system lymphoma. Acta Neuropathol.

[5] Braggio E, Van Wier S, Ojha J, McPhail E, Asmann YW, Egan J, da Silva JA, Schiff D, Lopes MB, Decker PA, Valdez R, Tibes R, Eckloff B (2015). Genome-wide analysis uncovers novel recurrent alterations in primary central nervous system lymphomas. Clin Cancer Res.

[6] Vater I, Montesinos-Rongen M, Schlesner M, Haake A, Purschke F, Sprute R, Mettenmeyer N, Nazzal I, Nagel I, Gutwein J, Richter J, Buchhalter I, Russell RB (2015). The mutational pattern of primary lymphoma of the central nervous system determined by whole-exome sequencing. Leukemia.

[7] Bruno A, Boisselier B, Labreche K, Marie Y, Polivka M, Jouvet A, Adam C, Figarella-Branger D, Miquel C, Eimer S, Houillier C, Soussain C, Mokhtari K (2014). Mutational analysis of primary central nervous system lymphoma. Oncotarget.

[8] Gonzalez-Aguilar A, Idbaih A, Boisselier B, Habbita N, Rossetto M, Laurenge A, Bruno A, Jouvet A, Polivka M, Adam C, Figarella-Branger D, Miquel C, Vital A (2012). Recurrent mutations of MYD88 and TBL1XR1 in primary central nervous system lymphomas. Clin Cancer Res.

[9] Sung CO, Kim SC, Karnan S, Karube K, Shin HJ, Nam DH, Suh YL, Kim SH, Kim JY, Kim SJ, Kim WS, Seto M, Ko YH (2011). Genomic profiling combined with gene expression profiling in primary central nervous system lymphoma. Blood.

[10] Nakamura T, Tateishi K, Niwa T, Matsushita Y, Tamura K, Kinoshita M, Tanaka K, Fukushima S, Takami H, Arita H, Kubo A, Shuto T, Ohno M (2016). Recurrent mutations of CD79B and MYD88 are the hallmark of primary central nervous system lymphomas. Neuropathol Appl Neurobiol.

[11] Yamada S, Ishida Y, Matsuno A, Yamazaki K (2015). Primary diffuse large B-cell lymphomas of central nervous system exhibit remarkably high prevalence of oncogenic MYD88 and CD79B mutations. Leuk Lymphoma.

[12] Montesinos-Rongen M, Godlewska E, Brunn A, Wiestler OD, Siebert R, Deckert M (2011). Activating L265P mutations of the MYD88 gene are common in primary central nervous system lymphoma. Acta Neuropathol.

[13] Wilson WH, Young RM, Schmitz R, Yang Y, Pittaluga S, Wright G, Lih CJ, Williams PM, Shaffer AL, Gerecitano J, de Vos S, Goy A, Kenkre VP (2015). Targeting B cell receptor signaling with ibrutinib in diffuse large B cell lymphoma. Nat Med.

[14] Bernard S, Goldwirt L, Amorim S, Brice P, Brière J, de Kerviler E, Mourah S, Sauvageon H, Thieblemont C (2015). Activity of ibrutinib in mantle cell lymphoma patients with central nervous system relapse. Blood.

[15] Jardin F (2014). Next generation sequencing and the management of diffuse large B-cell lymphoma: from whole exome analysis to targeted therapy. Discov Med.

[16] Schwarzenbach H, Hoon DS, Pantel K (2011). Cell-free nucleic acids as biomarkers in cancer patients. Nat Rev Cancer.

[17] Leary RJ, Sausen M, Kinde I, Papadopoulos N, Carpten JD, Craig D, O’Shaughnessy J, Kinzler KW, Parmigiani G, Vogelstein B, Diaz LA, Velculescu VE (2012). Detection of chromosomal alterations in the circulation of cancer patients with whole-genome sequencing. Sci Transl Med.

[18] Heitzer E, Ulz P, Geigl JB (2015). Circulating tumor DNA as a liquid biopsy for cancer. Clin Chem.

[19] Lebofsky R, Decraene C, Bernard V, Kamal M, Blin A, Leroy Q, Rio Frio T, Pierron G, Callens C, Bieche I, Saliou A, Madic J, Rouleau E (2015). Circulating tumor DNA as a non-invasive substitute to metastasis biopsy for tumor genotyping and personalized medicine in a prospective trial across all tumor types. Mol Oncol.

[20] Roschewski M, Dunleavy K, Pittaluga S, Moorhead M, Pepin F, Kong K, Shovlin M, Jaffe ES, Staudt LM, Lai C, Steinberg SM, Chen CC, Zheng J (2015). Circulating tumour DNA and CT monitoring in patients with untreated diffuse large B-cell lymphoma: a correlative biomarker study. Lancet Oncol.

[21] Hattori K, Sakata-Yanagimoto M, Okoshi Y, Goshima Y, Yanagimoto S, Nakamoto-Matsubara R, Sato T, Noguchi M, Takano S, Ishikawa E, Yamamoto T, Matsumura A, Chiba S (2017). MYD88 (L265P) mutation is associated with an unfavourable outcome of primary central nervous system lymphoma. Br J Haematol.

[22] Schwaederle M, Husain H, Fanta PT, Piccioni DE, Kesari S, Schwab RB, Banks KC, Lanman RB, Talasaz A, Parker BA, Kurzrock R (2016). Detection rate of actionable mutations in diverse cancers using a biopsy-free (blood) circulating tumor cell DNA assay. Oncotarget.

[23] Bettegowda C, Sausen M, Leary RJ, Kinde I, Wang Y, Agrawal N, Bartlett BR, Wang H, Luber B, Alani RM, Antonarakis ES, Azad NS, Bardelli A (2014). Detection of circulating tumor DNA in early- and late-stage human malignancies. Sci Transl Med.

[24] Boisselier B, Gállego Pérez-Larraya J, Rossetto M, Labussière M, Ciccarino P, Marie Y, Delattre JY, Sanson M (2012). Detection of IDH1 mutation in the plasma of patients with glioma. Neurology.

[25] Choi JJ, Reich CF, Pisetsky DS (2005). The role of macrophages in the in vitro generation of extracellular DNA from apoptotic and necrotic cells. Immunology.

[26] Jiang N, Reich CF, Pisetsky DS (2003). Role of macrophages in the generation of circulating blood nucleosomes from dead and dying cells. Blood.

[27] Hohaus S, Giachelia M, Massini G, Mansueto G, Vannata B, Bozzoli V, Criscuolo M, D’Alò F, Martini M, Larocca LM, Voso MT, Leone G (2009). Cell-free circulating DNA in Hodgkin's and non-Hodgkin's lymphomas. Ann Oncol.

[28] Baraniskin A, Kuhnhenn J, Schlegel U, Schmiegel W, Hahn S, Schroers R (2012). MicroRNAs in cerebrospinal fluid as biomarker for disease course monitoring in primary central nervous system lymphoma. J Neurooncol.

[29] Baraniskin A, Zaslavska E, Nöpel-Dünnebacke S, Ahle G, Seidel S, Schlegel U, Schmiegel W, Hahn S, Schroers R (2016). Circulating U2 small nuclear RNA fragments as a novel diagnostic biomarker for primary central nervous system lymphoma. Neuro Oncol.

[30] Frenel JS, Carreira S, Goodall J, Roda D, Perez-Lopez R, Tunariu N, Riisnaes R, Miranda S, Figueiredo I, Nava-Rodrigues D, Smith A, Leux C, Garcia-Murillas I (2015). Serial next-generation sequencing of circulating cell-free DNA evaluating tumor clone response to molecularly targeted drug administration. Clin Cancer Res.

[31] Camus V, Sarafan-Vasseur N, Bohers E, Dubois S, Mareschal S, Bertrand P, Viailly PJ, Ruminy P, Maingonnat C, Lemasle E, Stamatoullas A, Picquenot JM, Cornic M (2016). Digital PCR for quantification of recurrent and potentially actionable somatic mutations in circulating free DNA from patients with diffuse large B-cell lymphoma. Leuk Lymphoma.

[32] Jamal-Hanjani M, Wilson GA, Horswell S, Mitter R, Sakarya O, Constantin T, Salari R, Kirkizlar E, Sigurjonsson S, Pelham R, Kareht S, Zimmermann B, Swanton C (2016). Detection of ubiquitous and heterogeneous mutations in cell-free DNA from patients with early-stage non-small-cell lung cancer. Ann Oncol.

[33] Xu L, Hunter ZR, Yang G, Cao Y, Liu X, Manning R, Tripsas C, Chen J, Patterson CJ, Kluk M, Kanan S, Castillo J, Lindeman N (2014). Detection of MYD88 L265P in peripheral blood of patients with Waldenström's Macroglobulinemia and IgM monoclonal gammopathy of undetermined significance. Leukemia.

[34] Pertesi M, Galia P, Nazaret N, Vallée M, Garderet L, Leleu X, Avet-Loiseau H, Foll M, Byrnes G, Lachuer J, McKay JD, Dumontet C (2015). Rare circulating cells in familial Waldenström macroglobulinemia displaying the MYD88 L265P mutation are enriched by Epstein-Barr virus immortalization. PLoS One.

[35] Scherer F, Kurtz DM, Newman AM, Stehr H, Craig AFM, Esfahani MS, Lovejoy AF, Chabon JJ, Klass DM, Liu CL, Zhou L, Glover C, Visser BC (2016). Distinct biological subtypes and patterns of genome evolution in lymphoma revealed by circulating tumor DNA. Sci Transl Med.

[36] Kurtz DM, Green MR, Bratman SV, Scherer F, Liu CL, Kunder CA, Takahashi K, Glover C, Keane C, Kihira S, Visser B, Callahan J, Kong KA (2015). Noninvasive monitoring of diffuse large B-cell lymphoma by immunoglobulin high-throughput sequencing. Blood.

[37] Swerdlow SH, Campo E, Harris NL, Jaffe ES, Pileri SA, Stein H, Thiele J, Vardiman JW (2008). WHO Classification of Tumours of Haematopoietic and Lymphoid Tissues, Fourth Edition.

[38] Dubois S, Viailly PJ, Mareschal S, Bohers E, Bertrand P, Ruminy P, Maingonnat C, Jais JP, Peyrouze P, Figeac M, Molina TJ, Desmots F, Fest T (2016). Next generation sequencing in diffuse large B cell lymphoma highlights molecular divergence and therapeutic opportunities: a LYSA study. Clin Cancer Res.

[39] Li H, Durbin R (2009). Fast and accurate short read alignment with Burrows-Wheeler transform. Bioinformatics.

[40] Li H, Durbin R (2010). Fast and accurate long-read alignment with Burrows-Wheeler transform. Bioinformatics.

[41] Ning Z, Cox AJ, Mullikin JC (2001). SSAHA: a fast search method for large DNA databases. Genome Res.

[42] Boeva V, Popova T, Lienard M, Toffoli S, Kamal M, Le Tourneau C, Gentien D, Servant N, Gestraud P, Rio Frio T, Hupé P, Barillot E, Laes JF (2014). Multi-factor data normalization enables the detection of copy number aberrations in amplicon sequencing data. Bioinformatics.

[43] Bohers E, Viailly PJ, Dubois S, Bertrand P, Maingonnat C, Mareschal S, Ruminy P, Picquenot JM, Bastard C, Desmots F, Fest T, Leroy K, Tilly H (2015). Somatic mutations of cell-free circulating DNA detected by next-generation sequencing reflect the genetic changes in both germinal center B-cell-like and activated B-cell-like diffuse large B-cell lymphomas at the time of diagnosis. Haematologica.

[44] Poulain S, Boyle EM, Tricot S, Demarquette H, Doye E, Roumier C, Duthilleul P, Preudhomme C, Maurage CA, Morschhauser F (2015). Absence of CXCR4 mutations but high incidence of double mutant in CD79A/B and MYD88 in primary central nervous system lymphoma. Br J Haematol.

[45] Armbruster DA, Pry T (2008). Limit of blank, limit of detection and limit of quantitation. Clin Biochem Rev.

[46] Brennan CW, Verhaak RG, McKenna A, Campos B, Noushmehr H, Salama SR, Zheng S, Chakravarty D, Sanborn JZ, Berman SH, Beroukhim R, Bernard B, Wu CJ (2013). The somatic genomic landscape of glioblastoma. Cell.

