# Supplementary material for: Non-invasive detection of somatic mutations using next-generation sequencing in primary central nervous system lymphoma
Source: Oncotarget. 2017 Jun 1;8(29):48157–68. doi: 10.18632/oncotarget.18325 (PMC5564634; doi:10.18632/oncotarget.18325)
Supplement: Supplementary file 1 [file oncotarget-08-48157-s001.pdf]

Non-invasive detection of somatic mutations using next-generation sequencing in primary central nervous system lymphoma

SUPPLEMENTARY MATERIALS

Supplementary Table 1: Tumor DNA and circulating DNA sequencing results, n=25

See Supplementary File 1

Supplementary Table 2: The Lymphopanel

Gene	Transcript reference	Hotspot/exons partially sequenced	Chromosomal location	Size sequenced (bp)
B2M	NM_000633	Hotspots exons 1 & 2, exon 3	15q21.1	360
BCL2	NM_004048	Hotspot exon 2	18q21.33	585
BRAF	NM_004333	Exon 15	7q34	119
CARD11	NM_032415	Coiled-coil domain exons 4-9	7p22.2	1121
CD58	NM_001779	Exons 1-6	1p13.1	753
CD79A	NM_001783	ITAM domain exons 4 & 5	19q13.2	183
CD79B	NM_000626	ITAM domain exons 5 & 6	17q23.3	141
CDKN2A	NM_058197	Exons 1, 2A, 2B, 3, 4 & 5	9p21.3	1,737
CDKN2B	NM_004936	Exons 1A, 1B & 2	9p21.3	1289
CIITA	NM_000246	Exons 1-19	16p13.13	3393
CREBBP	NM_004380	Exons 1-31	16p13.3	7323
EP300	NM_001429	Exons 1-31	22q13.2	7245
EZH2	NM_001203247	SET domain, hotspots exon 16 & 18	7q36.1	177
FOXO1	NM_002015	Hotspots exon 1 & FH domain exon 2	13q14.11	780
GNA13	NM_006572	Exons 1-4	17q24.1	1134
ID3	NM_002167	Exons 1 & 2	1p36.12	360
IRF4/MUM1	NM_002460	Exons 2-9	6p25.3	1356
ITPKB	NM_002221	Exons 2-8	1q42.12	2830
KMT2D/MLL2	NM_003482	Exons 1-54	12q13.12	16614
MEF2B	NM_005919	Exons 2-9	19p13.11	1107
MFHAS1	NM_004225	Exons 1-3	8p23.1	3159
MYC	NM_002467	Exons 1-3	8q24.21	1365
MYD88	NM_001172567	Exons 2-5	3p22.2	587
NOTCH1	NM_017617	PEST domain exon 34	9q34.3	1488
NOTCH2	NM_024408	Exons 26-28 & 34 (HD/PEST domains)	1p12-p11.2	2091
PIM1	NM_001243186	Exons 1-6	6p21.2	942
PRDM1/ BLIMP1	NM_001198	Exons 1-7	6q21	2478
SOCS1	NM_003745	Exon 2	16p13.13	636
STAT6	NM_001178078	Exons 9-14 (DNA binding domain hotspot)	12q13.3	795
TCF3	NM_001136139	B-HLH domain of E47 isoform exons 17 & 18	19p13.3	370
TNFAIP3	NM_001270507	Exons 2-9	6q23.3	2373
TNFRSF14	NM_003820	Exons 1-8	1p36.32	852
TP53	NM_000546	Mutation hotspots exons 4-10	17p13.1	1004
XPO1	NM_003400	Exons 15-18	2p15	640

Supplementary Table 3: Dilution test for *MYD88* c.T778C

Experiment	Tested VAF	Number of mutated reads (c.T778C)	Number of all reads	VAF (%)	Interpretation
No 1	1%	491	64825	0.75%	**Pos**
	0.50%	47	8942	0.53%	**Pos**
	0.10%	11	10361	0.11%	**Pos**
	0.05%	15	16220	0.09%	**Pos**
	0%	5	19895	0.03%	**Pos**
No 2	1%	228	33807	0.67%	**Pos**
	0.50%	45	8910	0.50%	**Pos**
	0.10%	4	3739	0.11%	**Pos**
	0.05%	0	2200	0%	**Neg**
	0%	1	5034	0.02%	**Pos**
No 3	1%	279	24184	1.15%	**Pos**
	0.50%	42	9192	0.46%	**Pos**
	0.10%	0	3778	0%	**Neg**
	0.05%	3	2451	0.12%	**Pos**
	0%	2	5208	0.04%	**Pos**
No 4	1%	71	8102	0.88%	**Pos**
	0.50%	21	5109	0.41%	**Pos**
	0.10%	7	3046	0.23%	**Pos**
	0.05%	4	2330	0.17%	**Pos**
	0%	0	3625	0%	**Neg**
No 5	1%	62	10145	0.61%	**Pos**
	0.50%	15	2059	0.73%	**Pos**
	0.10%	0	1112	0%	**Neg**
	0.05%	1	416	0.24%	**Pos**
	0%	0	443	0%	**Neg**
No 6	1%	106	9578	1.11%	**Pos**
	0.50%	13	1821	0.71%	**Pos**
	0.10%	2	1165	0.17%	**Pos**
	0.05%	0	569	0%	**Neg**
	0%	0	956	0%	**Neg**
