# Supplementary material for: Non-invasive detection of somatic mutations using next-generation sequencing in primary central nervous system lymphoma
Source: Oncotarget. 2017 Jun 1;8(29):48157–68. doi: 10.18632/oncotarget.18325 (PMC5564634; doi:10.18632/oncotarget.18325)
Supplement: Supplementary file 2 [file oncotarget-08-48157-s002.docx]

**Supplementary Table 1: Tumor DNA and circulating DNA sequencing results, n=25**

| **Patient** | | **Gene** | **SNV** | **Tumoral DNA** | | |  | **Cell-free DNA** | | | |
| --- | --- | --- | --- | --- | --- | --- | --- | --- | --- | --- | --- |
| **No** | **Age** |  |  | **Percentage**  **of tumor cell** | **VAF (%)** | **Reads number^a^** |  | **VAF (%)** | **Reads number^a^** | **Interpretation** | **Concentration (ng/ml)** |
| #5 | 71 | *PIM1* | NM_002648:exon4:c.G421C-(p.E141Q) | 25-50% | 31.22 | 69/221 |  | 0 | 0/62281 | Neg | 130.4 |
|  |  | *KMT2D* | NM_003482:exon48:c.15724_15730del-(p.5242_5244del) | | 47.2 | 194/411 |  | 0 | 0/4 | Neg |  |
|  |  | *CD79B* | NM_000626:exon5:c.T586G-(p.Y196D) |  | 50.8 | 190/374 |  | 6.9 | 46/663 | Pos |  |
|  |  | *MYD88* | NM_002468:exon5:c.T778C-(p.L265P) |  | 91.12 | 359/394 |  | 11 | 414/3762 | Pos |  |
| #15 | 69 | *PRDM1* | NM_001198:exon2:c.258_265del-(p.86_89del) | >50% | 72.38 | 76/105 |  | 0 | 0/2953 | Neg | 45.6 |
|  |  | *MYD88* | NM_002468:exon3:c.G649T-(p.V217F) |  | 32.76 | 114/348 |  | <1 | 12/46617 | Neg |  |
|  |  | *TNFAIP3* | NM_006290:exon3:c.389delT-(p.L130fs) |  | 69.68 | 131/188 |  | 0 | 0/7044 | Neg |  |
|  |  | *KMT2D* | NM_003482:exon29:c.C6161T-(p.A2054V) |  | 41.53 | 233/561 |  | <1 | 21/32950 | Neg |  |
| #8 | 63 | *MYD88* | NM_002468:exon5:c.T778C-(p.L265P) | >50% | 28.48 | 94/330 |  | 12.2 | 3024/24793 | Pos | 46 |
|  |  | *TNFAIP3* | NM_006290:exon2:c.C133T-(p.R45X) |  | 46.69 | 162/347 |  | 9.4 | 933/9924 | Pos |  |
|  |  | *TNFAIP3* | NM_006290:exon6:c.965_969del-(p.322_323del) |  | 46.02 | 104/226 |  | 9.2 | 3083/33517 | Pos |  |
|  |  | *PIM1* | NM_002648:exon2:c.C187T-(p.P63S) |  | 43.38 | 59/136 |  | <1 | 28/3652 | Neg |  |
|  |  | *PIM1* | NM_002648:exon4:c.C316T-(p.L106F) |  | 55.56 | 25/45 |  | 15.3 | 1480/9649 | Pos |  |
|  |  | *PIM1* | NM_002648:exon4:c.C373T-(p.P125S) |  | 36.96 | 17/46 |  | 9.4 | 918/9737 | Pos |  |
|  |  | *PIM1* | NM_002648:exon4:c.C550T-(p.L184F) |  | 51.69 | 107/207 |  | 14 | 2930/20930 | Pos |  |
|  |  | *PIM1* | NM_002648:exon4:c.C587G-(p.T196S) |  | 50.95 | 107/210 |  | 14.1 | 2938/20909 | Pos |  |
|  |  | *PIM1* | NM_002648:exon4:c.G589A-(p.V197I) |  | 51.43 | 108/210 |  | 14.1 | 2948/20917 | Pos |  |
|  |  | *PIM1* | NM_002648:exon5:c.G706C-(p.V236L) |  | 45.45 | 40/88 |  | 9.5 | 283/2973 | Pos |  |
| #25 | 68 | *TCF3* | NM_001136139:exon17:c.T1670C-(p.V557A) | 25-50% | 26.25 | 21/80 |  | 0 | 0/4 | Neg | 76.4 |
|  |  | *TCF3* | NM_001136139:exon17:c.T1653A-(p.N551K) |  | 26.25 | 21/80 |  | 0 | 0/4 | Neg |  |
|  |  | *TNFAIP3* | NM_006290:exon2:c.C163T-(p.Q55X) |  | 45.57 | 108/237 |  | <1 | 24/56065 | Neg |  |
|  |  | *MYD88* | NM_002468:exon5:c.T778C-(p.L265P) |  | 67.74 | 147/217 |  | <0.5 | 15/9270 | Neg |  |
|  |  | *CD79B* | NM_000626:exon5:c.T586A-(p.Y196N) |  | 73.36 | 179/244 |  | <1 | 3/2952 | Neg |  |
| #21 | 77 | *KMT2D* | NM_003482:exon48:c.C14878T-(p.R4960X) | >50% | 42.98 | 153/356 |  | 0 | 0/329 | Neg | 49.6 |
|  |  | *CARD11* | NM_032415:exon5:c.G367A-(p.G123S) |  | 47.01 | 157/334 |  | 0 | 0/1327 | Neg |  |
|  |  | *MYD88* | NM_002468:exon5:c.T778C-(p.L265P) |  | 67.31 | 313/465 |  | <0.5 | 9/5993 | Neg |  |
| #11 | 78 | *EZH2* | NM_004456:exon16:c.T1936C-(p.Y646H) | >50% | 39.34 | 96/244 |  | 0 | 0/2332 | Neg | 92.8 |
|  |  | *MYD88* | NM_002468:exon3:c.C656G-(p.S219C) |  | 47.58 | 128/269 |  | <1 | 1/29508 | Neg |  |
| #16 | 57 | *CDKN2A* | NM_000077:exon2:c.G329A-(p.W110X) | >50% | 53.13 | 17/32 |  | 0 | 0/1 | Neg | 47.2 |
|  |  | *CD79B* | NM_000626:exon5:c.T586A-(p.Y196N) |  | 47.22 | 34/72 |  | 0 | 0/2279 | Neg |  |
|  |  | *BRAF* | NM_004333:exon15:c.A1781G-(p.D594G) |  | 24.56 | 83/338 |  | <1 | 7/5980 | Neg |  |
|  |  | *MYD88* | NM_002468:exon5:c.T778C-(p.L265P) |  | 43.48 | 70/161 |  | <0.5 | 11/9386 | Neg |  |
| #18 | 64 | *MEF2B* | NM_001145785:exon9:c.C1033T-(p.R345W) | >50% | 45.83 | 11/24 |  | 0 | 0/125 | Neg | 46.8 |
|  |  | *CARD11* | NM_032415:exon7:c.G1010A-(p.R337Q) |  | 35.37 | 29/82 |  | 0 | 0/133 | Neg |  |
|  |  | *MYD88* | NM_002468:exon5:c.T778C-(p.L265P) |  | 44.03 | 70/159 |  | <0.5 | 24/23286 | Neg |  |
|  |  | *CREBBP* | NM_004380:exon27:c.G4534A-(p.A1512T) |  | 52.04 | 153/294 |  | 55.4 | 3096/5588 | Pos |  |
| #13 | 49 | *GNA13* | NM_006572:exon1:c.C92T-(p.S31F) | >50% | 50 | 4/8 |  | <1 | 1/2863 | Neg | 37.3 |
|  |  | *B2M* | NM_004048:exon1:c.37_38del-(p.13_13del) |  | 60.78 | 31/51 |  | <1 | 4/14153 | Neg |  |
|  |  | *PRDM1* | NM_001198:exon2:c.C180G-(p.Y60X) |  | 69.62 | 55/79 |  | <1 | 1/22877 | Neg |  |
|  |  | *PRDM1* | NM_001198:exon2:c.G291C-(p.E97D) |  | 70 | 56/80 |  | <1 | 0/22887 | Neg |  |
|  |  | *SOCS1* | NM_003745:exon2:c.G374A-(p.S125N) |  | 61.46 | 59/96 |  | <1 | 11/13632 | Neg |  |
|  |  | *MYD88* | NM_002468:exon5:c.T778C-(p.L265P) |  | 60.09 | 134/223 |  | 0.6 | 107/17998 | Pos |  |
|  |  | *TNFAIP3* | NM_006290:exon8:c.2051_2052del-(p.684_684del) |  | 70.16 | 134/191 |  | 0 | 0/40307 | Neg |  |
| #28 | 53 | *MYD88* | NM_002468:exon5:c.T778C-(p.L265P) | >50% | 25.56 | 23/90 |  | 0 | 0/57 | Neg | 23.6 |
|  |  | *TP53* | NM_000546:exon7:c.G733A-(p.G245S) |  | 56 | 126/225 |  | <1 | 2/1475 | Neg |  |
| #22 | 53 | *GNA13* | NM_006572:exon1:c.A71G-(p.E24G) | >50% | 9.68 | 3/31 |  | - | - | - | 44 |
|  |  | *TP53* | NM_000546:exon8:c.G818A-(p.R273H) |  | 73.58 | 78/106 |  | <1 | 1/967 | Neg |  |
|  |  | *MYD88* | NM_002468:exon5:c.T778C-(p.L265P) |  | 43.92 | 159/362 |  | <0.5 | 1/737 | Neg |  |
| #24 | 74 | *ITPKB* | NM_002221:exon2:c.C334T-(p.Q112X) | >50% | 33.91 | 39/115 |  | 0 | 0/4 | Neg | 185.6 |
|  |  | *ITPKB* | NM_002221:exon2:c.G1527A-(p.W509X) |  | 32.95 | 58/176 |  | <1 | 1/1510 | Neg |  |
|  |  | *GNA13* | NM_006572:exon4:c.976delC-(p.L326fs) |  | 34.74 | 107/308 |  | <1 | 4/2753 | Neg |  |
|  |  | *PIM1* | NM_002648:exon3:c.C202T-(p.H68Y) |  | 31.76 | 74/233 |  | 0 | 0/30 | Neg |  |
|  |  | *ITPKB* | NM_002221:exon2:c.G260A-(p.G87D) |  | 33.19 | 77/232 |  | 0 | 0/4 | Neg |  |
|  |  | *ITPKB* | NM_002221:exon2:c.G466A-(p.A156T) |  | 39.18 | 105/268 |  | - | - | - |  |
| #1 | 63 | *B2M* | NM_004048:exon1:c.C7G-(p.R3G) | >50% | 27.97 | 73/261 |  | 0 | 0/13223 | Neg | 76.8 |
|  |  | *TNFAIP3* | NM_006290:exon4:c.634+2T>A |  | 65.08 | 192/295 |  | <1 | 1/20834 | Neg |  |
|  |  | *MYD88* | NM_002468:exon5:c.A880C-(p.T294P) |  | 39.15 | 287/733 |  | 0 | 0/117 | Neg |  |
| #12 | 61 | *IRF4* | NM_002460:exon2:c.A142G-(p.S48G) | >50% | 8.42 | 8/95 |  | - | - | - | 143.2 |
|  |  | *PIM1* | NM_002648:exon4:c.G403A-(p.E135K) |  | 42.86 | 15/35 |  | - | - | - |  |
|  |  | *BCL2* | NM_000633:exon2:c.C314A-(p.S105Y) |  | 24.79 | 29/117 |  | 0 | 1/26813 | Neg |  |
|  |  | *IRF4* | NM_002460:exon2:c.9_21del-(p.3_7del) |  | 100 | 25/25 |  | - | - | - |  |
|  |  | *IRF4* | NM_002460:exon2:c.G38C-(p.G13A) |  | 96.15 | 25/26 |  | - | - | - |  |
|  |  | *MYD88* | NM_002468:exon5:c.T778C-(p.L265P) |  | 47.5 | 76/160 |  | <0.5 | 64/38941 | Neg |  |
|  |  | *PIM1* | NM_002648:exon5:c.G677A-(p.W226X) |  | 65.79 | 75/114 |  | <1 | 1/623 | Neg |  |
|  |  | *KMT2D* | NM_003482:exon51:c.A16264G-(p.M5422V) |  | 29.26 | 139/475 |  | <1 | 16/18113 | Neg |  |
|  |  | *PIM1* | NM_002648:exon5:c.G784A-(p.E262K) |  | 49.14 | 172/350 |  | <1 | 5/17077 | Neg |  |
|  |  | *PIM1* | NM_002648:exon4:c.C550T-(p.L184F) |  | 54.09 | 172/318 |  | <1 | 30/35470 | Neg |  |
|  |  | *PIM1* | NM_002648:exon6:c.G877A-(p.V293I) |  | 43.79 | 254/580 |  | <1 | 4/8094 | Neg |  |
| #26 | 65 | *KMT2D* | NM_003482:exon51:c.A16264G-(p.M5422V) | >50% | 4.8 | 17/354 |  | - |  |  | 111.2 |
|  |  | *PIM1* | NM_002648:exon6:c.G877A-(p.V293I) |  | 7.22 | 14/194 |  | <1 | 3/7946 | Neg |  |
|  |  | *PIM1* | NM_002648:exon5:c.G677A-(p.W226X) |  | 9.76 | 12/123 |  | 0 | 0/903 | Neg |  |
|  |  | *PIM1* | NM_002648:exon5:c.G784A-(p.E262K) |  | 6.36 | 25/393 |  | <1 | 8/20026 | Neg |  |
|  |  | *PIM1* | NM_002648:exon4:c.C550T-(p.L184F) |  | 12.59 | 17/135 |  | 24.3 | 5995/24631 | Pos |  |
|  |  | *PIM1* | NM_002648:exon4:c.G403A-(p.E135K) |  | 66.96 | 150/224 |  | 16 | 2311/14482 | Pos |  |
|  |  | *MYD88* | NM_002468:exon5:c.T778C-(p.L265P) |  | 54 | 189/350 |  | 28.3 | 7091/25095 | Pos |  |
| #27 | 63 | *MYD88* | NM_002468:exon5:c.T778C-(p.L265P) | 25% | 15.6 | 17/109 |  | 0 | 0/76 | Neg | 26.4 |
| #32 | 87 | *KMT2D* | NM_003482:exon34:c.C8401T-(p.R2801X) | >50% | 51.72 | 30/58 |  | <1 | 6/6737 | Neg | 49.6 |
|  |  | *IRF4* | NM_002460:exon2:c.C63G-(p.N21K) |  | 37.4 | 46/123 |  | <1 | 4/2756 | Neg |  |
|  |  | *GNA13* | NM_006572:exon4:c.A748G-(p.S250G) |  | 90.2 | 46/51 |  | <1 | 4/9644 | Neg |  |
|  |  | *CD79B* | NM_000626:exon5:c.A587C-(p.Y196S) |  | 63.44 | 59/93 |  | 0 | 0/823 | Neg |  |
|  |  | *MEF2B* | NM_001145785:exon3:c.C70T-(p.R24W) |  | 40.6 | 108/266 |  | <1 | 31/57551 | Neg |  |
| #19 | 61 | *MYD88* | NM_002468:exon5:c.T778C-(p.L265P) | 25% | 26.58 | 21/79 |  | 0 | 0/8663 | Neg | 37.7 |
|  |  | *CD79B* | NM_000626:exon5:c.A587G-(p.Y196C) |  | 42.86 | 24/56 |  | 0 | 0/1460 | Neg |  |
|  |  | *EP300* | NM_001429:exon31:c.C6922T-(p.R2308C) |  | 29.05 | 70/241 |  | 6.3 | 901/14202 | Pos |  |
| #14 | 82 | *PIM1* | NM_002648:exon4:c.C577T-(p.L193F) | 25-50% | 56.9 | 33/58 |  | <1 | 1/1500 | Neg | 62.4 |
|  |  | *MYD88* | NM_002468:exon5:c.T778C-(p.L265P) |  | 67.27 | 37/55 |  | 0.9 | 6/669 | Pos |  |
|  |  | *CARD11* | NM_032415:exon7:c.G1010A-(p.R337Q) |  | 70.18 | 40/57 |  | 0 | 0 | Neg |  |
|  |  | *CARD11* | NM_032415:exon6:c.T770C-(p.I257T) |  | 64.23 | 88/137 |  | 0 | 0/19 | Neg |  |
| #44 | 60 | *PIM1* | NM_002648:exon4:c.G290C-(p.S97T) | >50% | 47.37 | 18/38 |  | 0 | 0/94 | Neg | 46.4 |
|  |  | *PIM1* | NM_002648:exon4:c.T328C-(p.F110L) |  | 48.72 | 19/39 |  | 0 | 0/97 | Neg |  |
|  |  | *PIM1* | NM_002648:exon4:c.C577G-(p.L193V) |  | 41.04 | 55/134 |  | <1 | 1/2355 | Neg |  |
| #40 | 82 | *PIM1* | NM_002648:exon4:c.G508T-(p.D170Y) | 25% | 11.4 | 22/193 |  | 0 | 0/36352 | Neg | 39.2 |
|  |  | *PIM1* | NM_002648:exon4:c.G248A-(p.G83D) |  | 70 | 14/20 |  | <1 | 3/2760 | Neg |  |
|  |  | *PIM1* | NM_002648:exon3:c.240+1G>T |  | 21.19 | 25/118 |  | <1 | 1/4556 | Neg |  |
|  |  | *PIM1* | NM_002648:exon4:c.C379T-(p.Q127X) |  | 96.3 | 26/27 |  | <1 | 2/599 | Neg |  |
|  |  | *PIM1* | NM_002648:exon4:c.300_301AA-() |  | 96.77 | 30/31 |  | <1 | 2/603 | Neg |  |
|  |  | *CD79B* | NM_000626:exon5:c.T586C-(p.Y196H) |  | 48.48 | 128/264 |  | <1 | 3/2948 | Neg |  |
|  |  | *CDKN2A* | NM_000077:exon2:c.C172T-(p.R58X) |  | 98.86 | 87/88 |  | 0 | 0 | Neg |  |
|  |  | *PIM1* | NM_002648:exon4:c.G403A-(p.E135K) |  | 87.85 | 159/181 |  | <0.5 | 113/35528 | Neg |  |
|  |  | *MYD88* | NM_002468:exon5:c.T778C-(p.L265P) |  | 97.43 | 341/350 |  | <1 | 19/12273 | Neg |  |
|  |  | *KMT2D* | NM_003482:exon31:c.7780dupC-(p.L2594fs) |  | 49.15 | 29/59 |  | - |  |  |  |
| #46 | 71 | *B2M* | NM_004048:exon1:c.T35A-(p.L12Q) | 25-50% | 56.97 | 94/165 |  | <1 | 1/14956 | Neg | 39.8 |
| #42 | 68 | *MYC* | NM_002467:exon2:c.C420G-(p.I140M) | >50% | 38.46 | 5/13 |  | 0 | 0/1137 | Neg | 37 |
|  |  | *MYC* | NM_002467:exon2:c.C393G-(p.I131M) |  | 42.86 | 6/14 |  | 0 | 0/1171 | Neg |  |
|  |  | *MYD88* | NM_002468:exon5:c.T778C-(p.L265P) |  | 24.1 | 60/249 |  | <0.5 | 42/38853 | Neg |  |
|  |  | *TNFAIP3* | NM_006290:exon4:c.C617T-(p.P206L) |  | 28.74 | 73/254 |  | <1 | 17/38971 | Neg |  |
|  |  | *CREBBP* | NM_004380:exon23:c.C3955T-(p.R1319X) |  | 41.96 | 60/143 |  | <1 | 5/5521 | Neg |  |
|  |  | *KMT2D* | NM_003482:exon31:c.6868_6871del-(p.2290_2291del) |  | 37.93 | 88/232 |  | <1 | 62/8858 | Neg |  |
|  |  | *MYC* | NM_002467:exon2:c.357_358CT-() |  | 82.41 | 89/108 |  | <1 | 100/108062 | Neg |  |
|  |  | *MYC* | NM_002467:exon2:c.G339C-(p.Q113H) |  | 89.25 | 83/93 |  | 0 | 0/8118 | Neg |  |
|  |  | *MYC* | NM_002467:exon2:c.G742T-(p.G248C) |  | 92.39 | 85/92 |  | <1 | 1/11318 | Neg |  |
|  |  | *MYC* | NM_002467:exon2:c.C781G-(p.P261A) |  | 93.41 | 85/91 |  | <1 | 2/12901 | Neg |  |
|  |  | *MYC* | NM_002467:exon2:c.G569A-(p.S190N) |  | 92.37 | 121/131 |  | <1 | 5/2259 | Neg |  |
|  |  | *MYC* | NM_002467:exon2:c.C535T-(p.P179S) |  | 92.37 | 121/131 |  | <1 | 2/2101 | Neg |  |
| #48 | 62 | *CD79B* | NM_000626:exon5:c.T586C-(p.Y196H) | >50% | 6.79 | 11/162 |  | 0 | 0/88 | Neg | 27.6 |
|  |  | *MYD88* | NM_002468:exon5:c.T778C-(p.L265P) |  | 7.58 | 21/277 |  | 0.87 | 2/231 | Pos |  |
| #2 | 67 | *-* | - | >50% | - | - |  | <0.5 | 9/14377 | Neg | 31.8 |
| ^a^ Number of mutated reads on all reads.  VAF; variant allele frequency. | | | | | | | | | | | |
